# Influence of Statistical Estimators of Mutual Information and Data Heterogeneity on the Inference of Gene Regulatory Networks

**DOI:** 10.1371/journal.pone.0029279

**Published:** 2011-12-29

**Authors:** Ricardo de Matos Simoes, Frank Emmert-Streib

**Affiliations:** Computational Biology and Machine Learning Lab, Center for Cancer Research and Cell Biology, School of Medicine, Dentistry and Biomedical Sciences, Queen's University Belfast, Belfast, United Kingdom; Dana-Farber Cancer Institute, United States of America

## Abstract

The inference of gene regulatory networks from gene expression data is a difficult problem because the performance of the inference algorithms depends on a multitude of different factors. In this paper we study two of these. First, we investigate the influence of discrete mutual information (MI) estimators on the global and local network inference performance of the C3NET algorithm. More precisely, we study 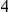 different MI estimators (Empirical, Miller-Madow, Shrink and Schürmann-Grassberger) in combination with 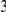 discretization methods (*equal frequency*, *equal width* and *global equal width* discretization). We observe the best global and local inference performance of C3NET for the Miller-Madow estimator with an *equal width* discretization. Second, our numerical analysis can be considered as a systems approach because we simulate gene expression data from an underlying gene regulatory network, instead of making a distributional assumption to sample thereof. We demonstrate that despite the popularity of the latter approach, which is the traditional way of studying MI estimators, this is in fact not supported by simulated and biological expression data because of their heterogeneity. Hence, our study provides guidance for an efficient design of a simulation study in the context of network inference, supporting a systems approach.

## Introduction

The mutual information (MI) is a measure to quantify the non-linear dependency between two random variables [1], [2]. The most popular strategies for estimating mutual information values are based on a discretized model for continuous data [3]. This strategy is widely known as the *histogram approach* that approximates the joint probability distribution by their empirical joint frequencies in bins of the two discretized random variables [1]. A variety of different mutual information estimators were developed in order to obtain statistical estimates for data sampled from an underlying distribution. The simplest estimator is the Empirical estimator [3] that is computed from the observed cell frequencies of a discretized distribution. However, it has been shown that the Empirical estimator underestimates the entropy due to an undersampling of cell frequencies and of zero cell frequencies which increase with the number of bins [4]. This is a major problem for practical applications due to finite data and the requirement for a large number of bins for accurate estimates. To account for the induced bias of the MI estimate, a variety of methods were developed that adjust the estimate by a constant factor [3], use a shrinkage regularization [5] or employ a Bayesian approach to estimate the joint frequencies for the bins from a Dirichlet distribution [6] to gain more accurate estimates.

In systems biology [7]–[10], many gene regulatory network (GRN) inference methods use mutual information as an estimator to unveil the interaction structure and the relations among genes in a cellular system from gene expression data [11]–[16]. One of the first methods based on mutual information for GRN inference was introduced in [17]. The underlying method, called *relevance network* (RN), assigns edges to gene pairs if the corresponding MI value is above a given threshold. The networks that result from application of RN are association networks because an edge between two genes indicates merely their association but not necessarily a causal effect [18]–[20]. A different type of GRN methods are network inference methods that aim to inferring causal interactions among genes and their products which can be experimentally validated [21]–[26]. The networks that result from such an inference are called *gene regulatory networks* [27]. So far, there is no generally agreed gold standard to conduct and include the routine of gene regulatory network inference and their analysis for molecular studies. However, necessary preprocessing steps of the data to prepare them for the subsequent inference of a gene regulatory network involve standardized procedures for the normalization of gene expression distributions within and between samples and a summarization step to obtain gene-centric values of the gene expression [28]–[30].

The major purpose of this paper is to investigate the influence of the MI estimator and the choice of the discretization method on the inference of gene regulatory networks. Despite the enormous popularity of the inference of GRNs this topic has so far only been addressed by a few studies. A notable exception is [31]. There gene regulatory network algorithms were evaluated for different mutual information estimators demonstrating that the choice of the estimator influences the inference performance in a significant manner. It has also been shown that the choice of the discretization and MI estimator is specific for a gene regulatory network inference method. In contrast to this study, there are many investigations that analyze statistical estimators of MI values directly without considering the MI estimator as part of a larger model, like the inference algorithm for a GRN. Usually, such studies assess either the estimates of the probability distribution respectively of the cell frequencies of an estimator, or study the resulting entropies [32]–[35]. The first approach is based on the *plug-in* usage of the estimated probabilities whereas the second utilizes the fact that the mutual information can be expressed in terms of entropies [36]. This can be seen as traditional approach because it places the statistical estimator itself in the focus of the investigation. In addition, another characteristics of these traditional studies is that they make assumptions about the distribution of the data and then assess the statistical MI estimator by means of simulated data, generated in accordance with these assumptions. This allows a thorough statistical analysis because each model parameter can be controlled appropriately, possibly, up to computational limitations.

The major purpose of this paper is to analyze the influence of statistical estimators of the mutual information on the inference of regulatory networks from large-scale gene expression data for the C3NET algorithm using global and local network-based error measures. More specifically, we investigate 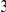 different discretization methods in combination with 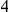 different statistical estimators for mutual information values. We infer networks from *in silico* (simulated) gene expression datasets generated for three Erdös-Rényi networks for various sample sizes and assess the influence of the MI estimators by global and local network-based error measures. In addition, we investigate and define data heterogeneity and discuss general consequences thereof on the simulation approach for network inference methods. This will provide us with general insights and reveals a problem of traditional studies of MI estimators which do not place the MI estimator into a model for which it is intended, but study it in isolation. For this reason, nonlinear effects that are only present for the larger model may lead to unexpected performance results which does not reflect the performance of the estimator in the isolated study. One reason therefore is the violation of assumptions. It is clear that assumptions that reflect real data *appropriately* lead to comparable results for simulated and real data. However, the more these assumptions are violated the more the results may deviate. This is well known, but does not reveal the only problem one encounters by using a statistical estimator within a larger model. The additional problem with the latter is in the specific context we are interested in, namely the inference of GRN from expression data, that the correlation structure within the data is only poorly understood, and, hence, there is no simple way known to simulate the expression of individual genes without simulating the entire gene network [37], [38]. In the results section of this paper we will show that as a consequence of these correlations among genes, there are many different types of probability distributions present within one gene regulatory network caused by the heterogeneity of the expression data. This makes it practically impossible to reduce the test of a MI estimator to a single probability distribution, but one would need to consider a *distribution* of probability distributions to test a MI estimator. From the presence of the data heterogeneity we conclude that a MI estimator can only be meaningfully studied within a network inference model and genome-wide data. This can be seen as a systems approach [39], [40] because reducing either the model to the MI estimator solely or the data to a *fixed* joint probability distribution does not lead to a realistic testing of the biological system.

This paper is organized as follows. In the next section we introduce the methods used for our analysis. Then we present numerical results of the influence different MI estimators have on the inference performance of C3NET by using global and local error measures. In addition, we analyze the effect of data heterogeneity on the estimation of MI values. The paper finishes with a discussion and conclusions.

## Methods

### The network inference method C3NET

The C3NET (conservative causal core) algorithm consists of three main steps [21]. The first step is for estimating mutual information values for all gene pairs. In the second step, the most significant link for each gene is selected. In the third step, non-significant links, according to a chosen significance level 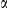, between gene pairs are eliminated by application of a multiple testing correction procedure. The complexity for multiple hypothesis testing (MHT) for C3NET is 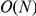, whereas 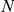 corresponds to the number of genes. The C3NET algorithm selects at most 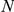 edges and therefore at most 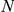 multiple tests are required. In comparison, other gene regulatory network inference approaches, e.g., RN [41], ARACNE [25] or CLR [42] eliminate non-significant links between *all* possible gene pairs in the first step. This leads to a complexity for multiple hypothesis testing (MHT) of 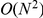. Some methods often circumvent the more extensive computational effort by applying arbitrarily chosen fixed significance thresholds.

**Figure 1:**
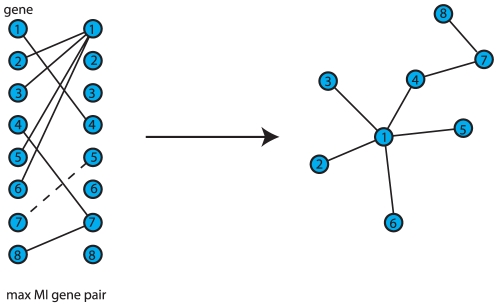
Principle working mechanism of C3NET. For each gene an edge is assigned to the gene neighbor with maximal MI value. The MI value between gene 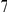 and 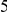 (dashed) is not significant. The resulting network describes the core of a gene regulatory network.

The inferred edges in a C3NET gene regulatory network correspond to the highest MI value among the neighbor edges for each gene. This implies that the highest possible number of edges that can be inferred by C3NET is equal to the number of genes under consideration. This number can decrease for several reasons. For example, when two genes have the same edge with maximum MI value. In this case, the same edge would be chosen by both genes to be included in the network. However, if an edge is already present another inclusion does not lead to an additional edge. Another case corresponds to the situation when a gene does not have significant edges at all. In this case, apparently, no edge can be included in the network. Since C3NET employs MI values as test statistics among genes, there is no directional information that can be inferred thereof. Hence, the resulting network is undirected and unweighted. Figure 1 shows the principle working mechanism of the network inference method C3NET.

### Estimating mutual information

In the following we investigate 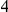 different types of estimators that are based on the so called histogram approach. In the first step the expression values of two genes are discretized into defined intervals, denoted as bins. The mutual information is a measure for the nonlinear dependence of the two random variables. Mutual information is defined by the marginal probability 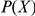 and 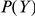 and joint probability 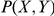 of two random variables 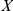 and 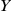 [36]:
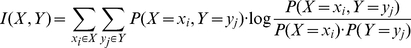
(1)Here 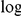 means the logarithm to the base of 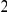. 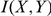 is always 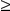
0. For example if the two random variables are independent from each other the mutual information is 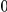, because 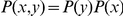.

The mutual information can also be expressed in terms of entropies [36],
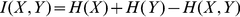
(2)Here the entropy for a random variable 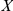 is defined by:
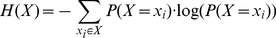
(3)and the joint entropy 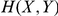 is given by
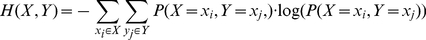
(4)We describe four different strategies for estimating mutual information for a discretized model. The simplest estimator is the empirical MI estimator [3] that estimates entropy from the observed joint frequencies for each bin. The empirical entropy 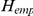 is estimated from the observed probability distribution with 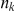 number of samples in bin 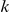, total number of samples 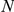 and the total number of bins 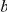. Note that the entropy formulas shown in the following are for a single random variable.
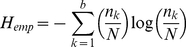
(5)


The Empirical estimator gives the maximum-likelihood entropy estimate for a discretized random variable. A main problem of the empirical approach is the underestimation of the true entropy 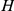 due to undersampling of the cell frequencies with increasing number of bins. A variety of approaches were developed to account for the induced bias that range from correcting the estimate by a constant factor or using a multinomial distribution to model the extend of missing information. In the following we show 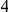 different MI estimators that are based on a discretized model.

The Miller-Madow estimator [3] accounts for the undersampling bias by adjusting the estimate by a constant factor that is proportional to the bin size and sample size:
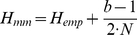
(6)for 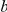 number of bins and 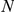 number of samples.

The following two estimators consider the correction of the probability distributions directly. The shrinkage estimator [5] combines two models defining a model with cell frequency of 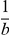 and a model defining the empirical cell frequency for each bin 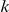.
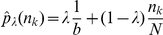
The weighting parameter 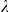 is estimated by minimizing the mean squared error for the two models for each 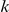 of 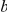 bins (Equation 7).
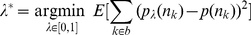
(7)The entropy for the shrinkage optimized probability distribution is computed by:
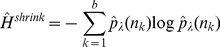
(8)


The Schürmann-Grassberger estimator [6] is based on a Bayesian approach that uses the Dirichlet probability distribution as conjugate prior for the likelihood given by the Empirical estimator. The Dirichlet distribution describes the distribution of probability distributions with mean 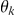.
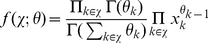
(9)


The mean 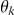 probability for each bin 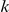 is estimated from the posterior using the Schürmann-Grassberger parameter 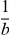 that equals:
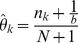
(10)In overall one pseudocount is added to the total sample count 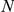. The entropy is estimated by:
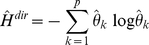
(11)


The MI estimators are used in combination with three different discretization methods. The first, *equal frequency* method assigns the same frequency of values to each bin 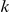. The *equal width* method uses for each interval width for each bin the same value. However, this is done independently for each of the two random variables 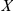 and 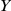. In contrast, the *global equal width* uses the same interval width for both random variables. The number of bins are defined by the proportional 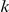-interval discretization method with 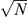 [43], where the number of bins is dependent on the number of samples.

### Global error measure

In order to measure the influence of the MI estimators on the inference performance of the C3NET algorithm we use the area under the precision-recall curve for the *receiver operator characteristics* (AUC-PR) [44]. The precision and recall measures [45] are obtained by comparison of an inferred (predicted) network with the true network used to simulate the underlying data. The recall, also known as the sensitivity, denotes the proportion of true positive edges relative to all edges in the reference network.
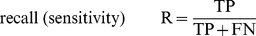
(12)


The precision gives the proportion of correctly inferred edges relative to all inferred edges.
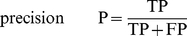
(13)


The predicted edges of gene regulatory network are ranked, e.g., by their respective MI estimate or alternative statistical measures. For a given threshold 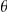 a confusion matrix can be defined when the true underlying network is known such as for simulated data. A confusion matrix tabulates the number of true positive, false positives, true negatives and false negative predictions. The PR-curve describes the precision (predicted true positives) as function of the sensitivity (recall, true positive rate) obtained by using various threshold values 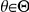 for the rank measures of the predicted edges. The AUC-PR area under the curve value is computed by a numerical integration along each point of the curve.

### Ensemble data and local network-based measures

In contrast to the above measure, which is a global error measure, we use also local network-based measures to assess the influence of the MI estimators. The principle idea of local *network-based measures* was introduced in [38], [46]. These local network-based error measures are based on ensemble data and the availability of a reference network 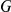 that represents the true regulatory network. Ensemble data means that there is more than one dataset available from the biological phenomenon under investigation. This ensemble of data could be either obtained by bootstrapping from one large data set, or from a simulation study, or from multiple experiments.

**Figure 2:**
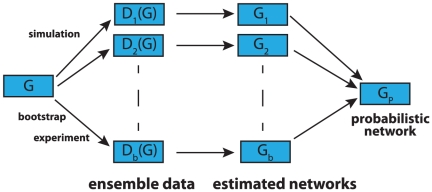
Schematic visualization of ensemble data from which networks are inferred and subsequently aggregated to estimate a probabilistic network.

After obtaining the ensemble of data 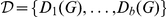, the inference algorithm is applied to 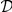 resulting in an ensemble of estimated networks 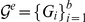 (see Fig. 2 for a visualization). Here we emphasize that each dataset depends on the underlying network structure, 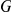, that governs the coupling among the genes by writing, e.g, 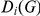. Further, this indicates that always the same network 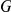 is used.

**Figure 3:**
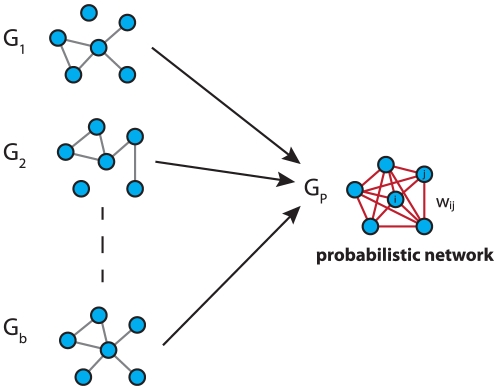
The ensemble of networks 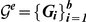 is used to obtain a weighted network 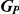.

If the network structure of the underlying network 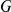 is available it is possible to obtain estimates of the TPR (true positive rate) of edges and non-edges in 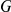. From the ensemble of estimated networks 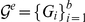 one obtains a probabilistic network 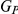. The edge weights of 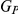 give the TPR for each edge which quantifies how often an edge was observed in the ensemble. The edge weights between gene 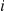 and 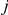 of 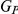 are defined by
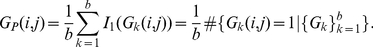
(14)The indicator function 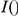 is 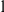 if an edge between gene 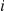 and 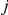 is observed in a network 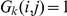 and 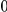 otherwise. From Eqn. 14 follows that 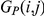 corresponds to the probability that an edge is present in 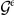 connecting gene 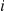 with 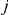. The combination of the networks in 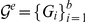 leading to the probabilistic network 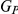 is visualized in Fig. 3.

### Local network-based error measure

We study the influence of the MI estimator on the inference performance for two classes of edges. The first class (Class I) of edges corresponds to linearly connected nodes and the second class (Class II) of edges corresponds to nodes with a high degree. The two edge classes are defined via *local network-based* measures. We define the *local network-based* measure 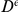 for each edge in an undirected graph, by the sum of the degrees of node 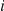 and the degrees of node 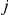:
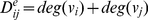
(15)Based on the values of 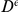 we define a binary classification for the edges by:

- Class I: edges with 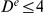 (corresponds to a chain-like structure)
- Class II: all other edges

**Figure 4:**
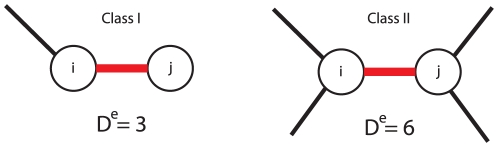
An edge (red) is scored according to the degree of the parental nodes of an undirected network (sum of degree 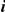 and degree 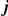) and assigned to Class I if 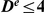 and to Class II if 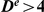.

In order to visualize our definition, we present in Fig. 4 two examples for 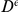. The left side shows an example for a Class I edge (with a score 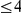) and the right side an example for a Class II edge (with a score 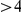). As described in the section ‘Ensemble data and local network-based measures’, a TPR is obtained for each edge. From the ensemble of datasets the distribution of the average TPRs is obtained for the two edge classes which we use for their comparison.

### Simulation of gene expression data

In order to study the influence of the network connectivity on the MI value estimators we are using random networks with different values of 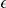. Here 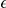 is the probability for the presence of an edge between two nodes [47]. Because real gene networks, e.g., the transcriptional regulatory network or the protein interaction network, are sparsely connected, the value of 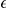 needs to be chosen to fall within a realistic interval. Typically, gene networks have an edge density of about 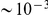 [48].

**Figure 5:**
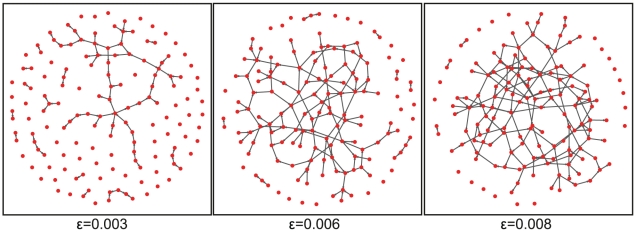
Shown are three Erdös-Rényi networks with edge density 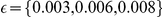.

We generate three Erdös-Rényi graphs [47], [49] with 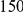 genes and an edge density of 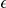 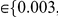 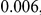 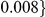. The resulting networks have 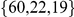 unconnected genes to model non-expressed genes. These three networks are shown in Figure 5. For our study we generate *steady-state* gene expression data using SYNTREN [50]. For each Erdös-Rényi network and sample size 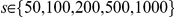 we generate 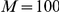 datasets.

## Results

In the following we study 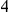 MI estimators in combination with 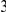 discretization methods. We assess the influence of these estimators on the inference of regulatory networks obtained with the C3NET algorithm by application of global and local network-based error measures. To ensure that our results are statistically robust, we base our study on ensemble data by simulating 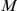 datasets for each studied condition. Using simulated data enables the comparison of the inferred networks with the true reference networks and also the control of important parameters. In the last results section we study the influence of data heterogeneity on the MI estimates.

### Influence of MI estimators on the global error measure AUC-PR

**Figure 6:**
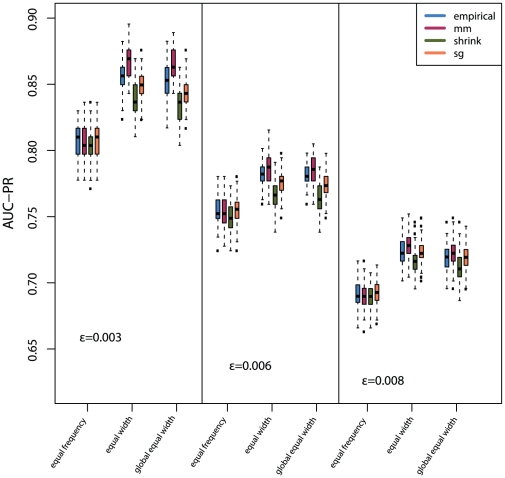
The influence of different discretization methods on the global network inference performance (AUC-PR) for three Erdös-Rényi networks and 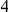 MI estimators. The simulated gene expression datasets have sample size 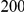.

First, we compare the impact of the discretization method on the AUC-PR (Figure 6). For all three Erdös-Rényi networks, the *equal width* and the *global equal width* discretization lead to a better inference performance of C3NET compared to the *equal frequency* discretization (Figure 6). Further, the *equal width* and *global equal width* discretization in combination with the Miller-Madow estimator is better than any other combination of MI estimator and discretization method. The second best MI estimator is the Empirical estimator. The Schürmann-Grassberger estimator and the Shrink estimator perform worse, whereas the Schürmann-Grassberger estimator performs better than the Shrink estimator (Figure 6). When using the *equal frequency* discretization, all MI estimators perform equally and show no substantial difference, see Fig. 6. Further, one can see that the network inference performance is decreasing with an increasing edge density. This is reasonable and related to the working mechanism of C3NET. Due to the fact the C3NET allows each gene to contribute at most one edge to the resulting network, the inference of networks that have a higher edge density is systematically disfavored.

**Figure 7:**
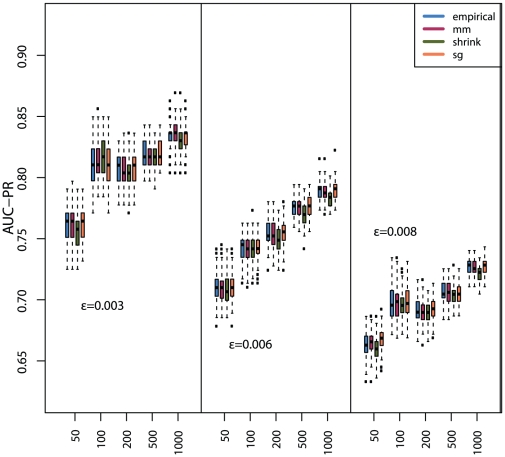
The influence of the *equal frequency* discretization method on the global network inference performance (AUC-PR) for three Erdös-Rényi networks and 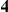 MI estimators.

**Figure 8:**
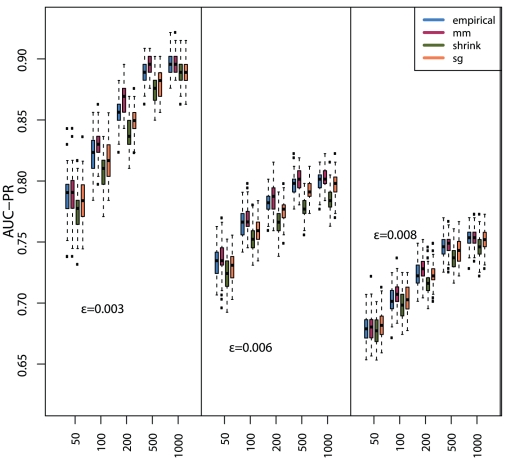
The influence of the *equal width* discretization method on the global network inference performance (AUC-PR) for three Erdös-Rényi networks and 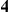 MI estimators.

**Figure 9:**
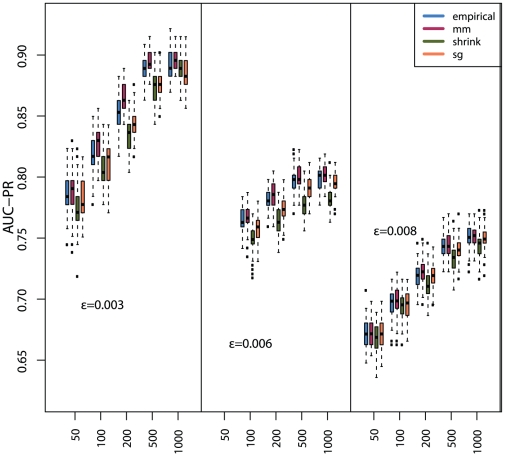
The influence of the *global equal width* discretization method on the global network inference performance (AUC-PR) for three Erdös-Rényi networks and 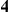 MI estimators.

In addition, we study also the dependency of the MI estimators, for each of the 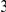 discretization methods, on the sample size. The corresponding results are shown in Fig. 7 (*equal frequency* discretization) Fig. 8 (*equal width* discretization) and Fig. 9 (*global equal width* discretization). For all investigated network types the inference performance increases with the sample size, as expected. However, independent of the influence of the sample size, the Miller-Madow estimator in combination with the *equal width* or the *global equal width* discretization show the best performance with respect to the inference performance of C3NET.

### Influence of MI estimators on the local error measure 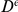


In the previous section we studied MI estimators by using a global error measure. When a global error measure is used, we actually do not obtain information about the performance of individual edges, but only of the average performance of all edges in the network. In order to *zoom in* to local properties of the network, we study in this section the local network-based measure 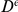. This measure allows to divide the edges in a network into two classes, according to the structural neighborhood of an edge, as defined in section ‘Local network-based error measure’. Specifically, this will allow us to gain information about the influence the MI estimators have on edges with a certain structural property. We use the local network-based measure 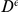 to distinguish between two edge classes. The first class represents edges from linearly connected genes (Class I) and the second class represents edges that belong to genes with multiple edges (Class II), see Fig. 4 for a visualization. In the following, we study the influence of the MI estimators on these two edge classes separately. We expect that edges from linear connected genes have only few dependencies that affect the underlying gene expression patterns and thus are more easier to infer. In contrast, edges connected to genes that are influenced by multiple other genes are expected to show more complex gene expression patterns and are therefore more difficult to estimate.

**Figure 10:**
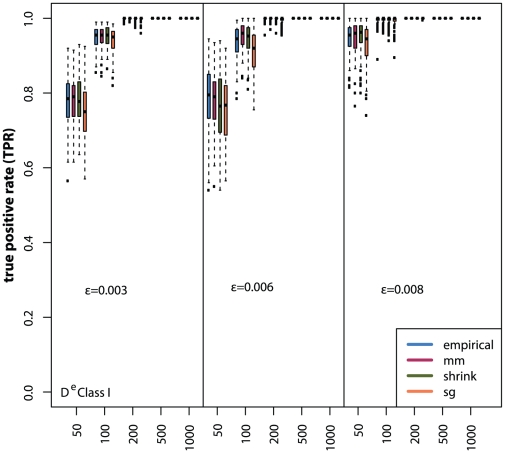
Local network inference performance for Class I edges. Simulated gene expression datasets for Erdös-Rényi networks with edge density 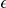 for sample sizes ranging from 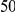 to 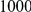 samples.

**Figure 11:**
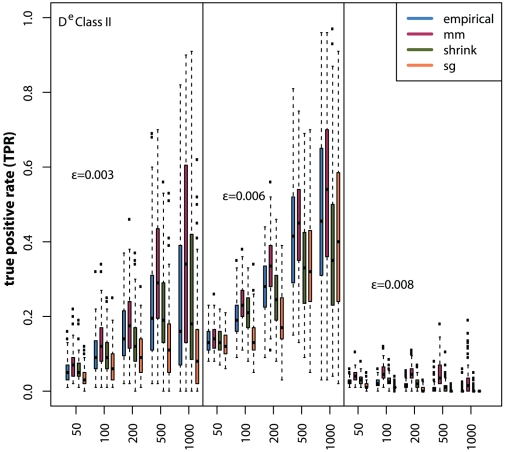
Local network inference performance for Class II edges. Simulated gene expression datasets for Erdös-Rényi networks with edge density 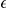 for sample sizes ranging from 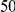 to 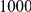 samples.

For the following simulations we use the *equal width* discretization in combination of the 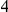 MI estimators for gene regulatory network inference with C3NET. In Figure 10 and 11 we show the distributions of true positive rates for the three random networks and different sample sizes for the two edge classes. In general, the Class I edges show a much better inference performance compared to the Class II edges. Also, for the Class I edges the estimators do not have a substantial influence on the inference performance. However, for the Class II edges we observe that the Miller-Madow estimator performs best, followed by the Empirical estimator. The Schürmann-Grassberger estimator and the shrinkage estimator rank last. In contrast to the global error measure (AUR-PR), for edges of Class II the shrinkage estimator performs better than the Schürmann-Grassberger estimator (Figure 11).

### Influence of data heterogeneity

Traditionally, when MI estimators are investigated, they are studied by making an assumption about the joint probability distribution 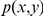 of the two random variables the MI estimator depends on. From this, the marginal distributions 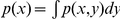 and 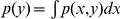 are obtained. Frequently, normality is assumed but also other probability distributions have been studied [32]–[34]. Regardless of the actual probability distribution selected, all studies have in common to make an implicate assumption which translates into a *homogeneity* of the data. That mean a MI estimator is investigated with respect to a *fixed* probability distribution from which data are sampled. This may be repeated for several different but *fixed* probability distributions. The crucial point here is that this investigation is done for each probability distribution separately which means that a MI estimator is assessed by using datasets that come from only one underlying distribution. With respect to this distribution, the sampled datasets are homogeneous because no otherwise distributed data are present. In this section we will study different aspects of the homogeneity assumption and consequences thereof. We proceed by, first, investigating various tests about the normality of the data and then consider arbitrarily distributed data.

We start by testing the null hypothesis if a gene expression profile is normally distributed, by using the Anderson-Darling test [51]. For the 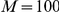 simulated gene expression datasets, we tested the null hypothesis of normality for the expression profiles. For the three Erdös-Rényi networks with edge density 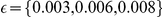 containing 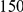 genes, 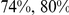 and 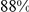 of the genes reject in average the null hypothesis testing for normality. These values are obtained for a significance level of 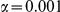 and a Bonferroni correction [52]. We repeated the same analysis for a normalized gene expression dataset from *S. cerevisiae* [42] containing 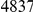 genes and 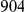 samples to see if our simulated data represent realistic aspects of biological data. Testing each of the 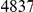 genes for normality leads to a rejection of 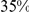 of the tests (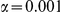, Bonferroni corrected). This demonstrates, first, that the characteristics of our simulated data is comparable to that of biological data and, second, that there is a non-negligible fraction of genes whose expression is not normally distributed, even after appropriate normalization of the data.

Next, we investigate the relation between the occurrences of TP and FP edges and the normality of the gene expression values. From testing the null-hypothesis that a gene expression profile follows a normal distribution, using the Anderson-Darling normality test, we obtain p-values for all genes. Combining pairs of p-values with Stouffer's method [53] gives us a p-value we assign to all gene pairs which correspond to edges and non-edges in the reference network. More precisely, we test the null hypothesis for normality of the gene expression profile of a gene using the Anderson-Darling test [51], as before. Then for each gene pair, their p-values are combined using the Stouffer method [53]. This method, first, transforms individual p-values into z-scores, 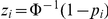. Here 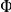 is the cumulative distribution function of the standard normal distribution. After this transformation the resulting z-scores are aggregated into a combined z-score, 
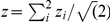
, from which the combined p-value is obtained. These combined p-values reflect the normality of the genes that enclose an edge (or a non-edge). We estimate these p-values for each of the 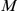 datasets. This results in 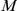 vectors of length 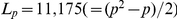 of p-values, which we rank in ascending order. We call these vectors 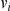, for 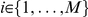. From the comparison of the inferred network with the reference network we obtain for each dataset a categorization of these p-values into the four categories TP, FP, TN and FN with respect to correctly/falsely identified edges. Considering TP and FP edges only, we obtain two categories which we use in the following. Due to the fact that, usually, the number of 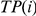 edges is not equal to the number of 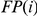 edges for a given dataset 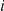, we identify their common length,
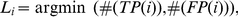
(16)and use exactly 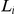 true positive and false positive hits from this dataset only. This information is used to define two rank vectors 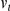 and 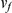, each of length 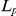, one for TP and one for FP edges, by extracting the ranks for the first 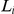 TP and FP edges for the 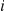-th dataset from the vector 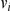. This results in two 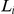 dimensional vectors 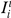 and 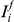 containing the ranks of the TP and FP edges in 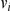. Starting from 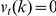 and 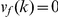 for 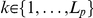, 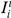 and 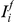 are used to update these vectors for every dataset by 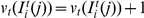 and 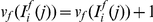 for 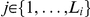. Repeating this procedure for all 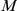 datasets provides us with two vectors, 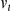 and 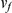, whose components reflect the frequency with which they occurred in all datasets. For example, 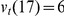 would mean that the edge ranked at position 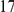 appeared 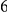 times in the set of TP edges among the first 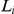 hits in the 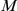 datasets that were considered. The interpretation for 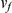 for the FP edges is analogously. For each set of datasets with sample size 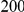 of the Erdös-Rényi network with 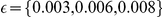, we compare the ranks of the p-values between TP edges, denoted by 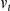, and FP edges denoted by 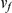.

**Figure 12:**
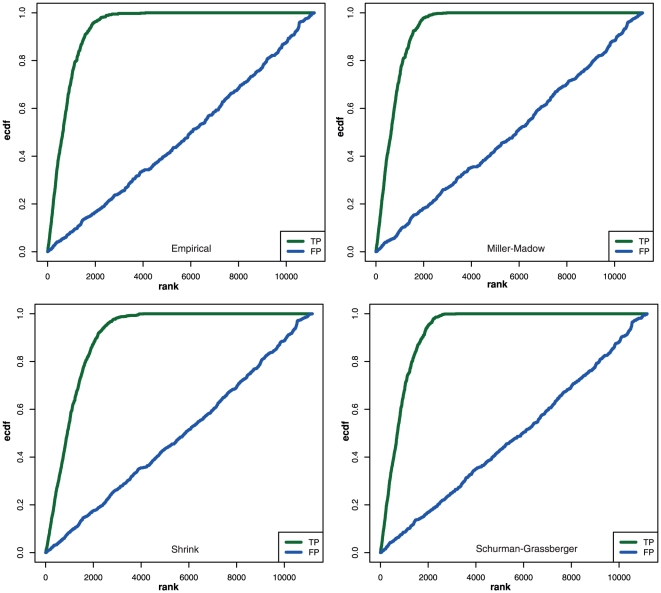
Empirical cumulative distribution functions (ecdf) for the four MI estimators. The green curves correspond to TP edges and the blue curves to FP edges as a function of their rank.

In Figure 12 we show the empirical cumulative distribution functions (ecdf) for 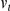 and 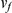 calculated for a Erdös-Rényi network with 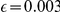. One can see that the TP edges are more likely to occupy lower ranked p-values compared to the FP edges. Hence, the distributions corresponding to TP edges have the tendency to show a stronger deviation from normality. In contrast, the curves of the FP edges follow a straight line which indicates the absence of a systematic relation between FP edges and the normality of the data. For the networks with 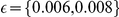 we obtain qualitatively similar results.

Finally, we generalize the above analysis by testing for general probability distributions, to see if there is a systematic relation between the occurrences of TP and FP edges and the distribution of the gene expressions. For this analysis we use a Kolmogorov-Smirnov test to compare the gene expression distributions for all edge and non-edge gene pairs. For a significance level of 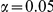 (Bonferroni corrected) the equality of the expression distribution was rejected for 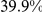, 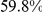 and 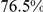 (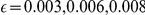) of all gene pairs. For reasons of comparison, we performed a similar analysis for the gene expression dataset from yeast [42] and found that 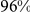 of the tests were rejected. Then we compared the ranks obtained from the Kolmogorov-Smirnov tests between TP and FP edges, as explained above for the normality test. Again, this procedures was performed for the Empirical, Miller-Madow, Shrinkage and Schürmann-Grassberger estimator for each expression dataset with sample size 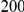 of the Erdös Rényi networks with 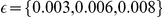. Similar to the results shown in Figure 12, we find that gene pairs of TP edges are likely to show a more prominent difference between their underlying expression profile (not shown). Hence, this observation is in agreement with the rank comparisons of TP and FP edges obtained from the Anderson-Darling normality test, and demonstrate that TP and FP edges behave quantitatively different, independent of distributional assumptions.

**Figure 13:**
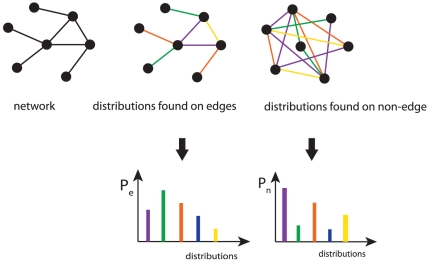
Schematic summary of the effect of data heterogeneity. Top row: Shown is a gene regulatory network (left) and the occurrence of different probability distributions observed on edges (middle) and non-edges (right). Bottom row: From the occurrence frequency of different probability distributions the two discrete distributions 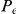 and 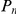 are obtained.

In Fig. 13 we summarize our findings about the data heterogeneity graphically. We found that for a given gene regulatory network one can find a multitude of different joint probability distributions, visualized by the different edge colors. Similarly, for gene pairs that are not directly connected by an edge (non-edges) one can also find many different probability distributions. From this one can obtain (discrete) probability distributions of the occurrence of probability distributions, visualized in the bottom part of Fig. 13. In these two diagrams, each color bar represents one specific probability distribution that can be found for the edges or non-edges. These discrete probability distributions 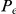 and 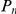 can even be different from each other.

Mathematically, the distributions 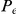 and 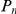 can be used to define the terms data heterogeneity respectively data homogeneity. More precisely, if we would observe 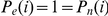 for one distribution 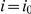, and 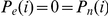 for all other distributions (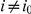), the underlying data would be homogeneous because they can be described by the probability distribution 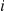. For all other distributions of 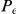 and 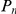 the data show, at least to some degree, a heterogeneity. It is easy to see that the case
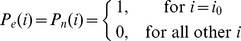
(17)corresponds to the conventional (implicit) assumption made when MI estimators are studied in isolation.

## Discussion

In this study we presented a comprehensive investigation of the influence the MI estimators have on the inference performance of C3NET. We observed a strong influence of the MI estimators and the discretization methods on the inference performance, revealed by global and local network-based error measures. In summary, we found that the Miller-Madow estimator in combination with the *equal width* and the *global equal width* discretization methods provided the best performance. However, the major influence on the C3NET inference performance was observed for the discretization method itself, whereas the *equal width* and the *global equal width* lead to significantly better results than the *equal frequency* discretization method. Hence, using the *equal frequency* discretization in applications is likely to lead to a reduced performance of C3NET because the inference performance is prominently lower compared to the other two discretization methods. A potential explanation why the Miller-Madow estimator performs better than the other estimators is that the Miller-Madow estimator is the only estimator among the used MI estimators that considers a bias correction, which depends on the sample size and the number of bins that are introduced by the discretization.

In [31] it was shown that the influence of the discretization method and a MI estimator is method specific, e.g., for ARACNE, CLR and MRNET. In their study, MRNET and ARACNE performed best with the *equal frequency* discretization in combination with the Empirical or Miller-Madow estimator while CLR was observed to be less influenced by different estimators. In contrast to our results for C3NET, the *equal width* discretization method was observed to outperform the *equal frequency* discretization method if used in combination with the Miller-Madow estimator. A major factor that explains the differences of the discretization methods on the network inference performance of our study compared to the study performed by [31] are likely due to the different working mechanisms and characteristics of the statistical principles, employed by the different network inference methods. For this reason it is necessary to identify the optimal combination of a statistical MI estimator and a discretization method for each network inference algorithm individually, as pointed out in [31].

Interestingly, the influences of the different MI estimators found in [31] are less pronounced than the one we found for the C3NET algorithm. A reason for this may be that the ensemble data used in [31] consist of only 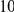 expression datasets for each setting. A large ensemble size of 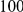 datasets, as used in our study, allows to capture finer variations among the simulated datasets and thus allows more robust comparisons.

Another factor that could lead to differences, is the simulation strategy used for the simulation of gene expression data. For example, the effect of noise and missing data were studied in [31] and it was shown that this is an important influence that needs to be taken into account when assessing the inference performance. However, future studies are necessary to investigate this influence in more detail and also to provide guidance with respect to the selection of a simulation setting.

Global error measures make the implicit assumption to observe an approximately equal inference performance for all edges in the network. However, for C3NET it was shown that edges of linearly connected nodes, e.g., edges of leaf nodes, have a higher inference performance than edges of highly connected nodes [21]. Hence, it is likely that edges of highly connected genes are more difficult to infer due to the more complex expression patterns of the corresponding genes. For this reason, we used in addition to global error measures also local network-based error measures to study the inference performance for edges of linearly connected genes (Class I) and all other edges (Class II). We found that edges from genes with a high degree (Class II) are likely to be underrepresented in the inferred networks because they have a lower (median) true positive rate than edges from Class I. Further, we compared the influence of the MI estimator on the edge classes Class I and Class II. Among the tested combinations of discretization methods and estimators, the Miller-Madow estimator with *equal width* or *global equal width* discretization showed the best performance on the inference performance of C3NET, independent from the edge density and the sample size.

In order to obtain the above results we simulated gene expression data from underlying regulatory networks, instead of making a distributional assumption to sample data from such a distribution. The latter is the traditional approach to study MI estimators [32]–[34]. Despite its popularity and simplicity we demonstrated that in the context of the inference of GRNs this reductionistic approach is not appropriate because of the data heterogeneity. Specifically, we showed that one can find a multitude of different probability distributions in simulated as well as biological expression data which can be represented as a (discrete) distribution of probability distributions, 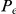, for edges and, 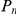, for non-edges. Hence, assuming the presence of merely one probability distribution is not supported by data. For this reason, if one would like to study MI estimators in isolation, one would need to make assumptions not only about the usage of one probability distribution, but of the distribution of probability distributions (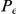, for edges and, 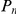 for non-edges). In order to avoid this complication, we recommend to simulate expression data from an underlying gene regulatory network because this provides naturally such a distribution of probability distributions.

In summary, we studied the influence of discrete MI estimators and discretization methods on the inference performance of C3NET and provided suggestions for the most beneficial combination. However, our study may be also useful for the development of novel MI estimators that take the various underlying probability distributions for different edge classes into consideration. Future studies are required to evaluate the vast catalogue of existing and novel MI estimators, the impact of different network structures and the simulation procedures in order to explore the particular factors that are required to understand the influence of different MI estimators on individual inference algorithms to enable an efficient analysis of real biological gene expression datasets.
